# Antibody evasion properties of SARS-CoV-2 Omicron sublineages

**DOI:** 10.1038/s41586-022-04594-4

**Published:** 2022-03-03

**Authors:** Sho Iketani, Lihong Liu, Yicheng Guo, Liyuan Liu, Jasper F.-W. Chan, Yiming Huang, Maple Wang, Yang Luo, Jian Yu, Hin Chu, Kenn K.-H. Chik, Terrence T.-T. Yuen, Michael T. Yin, Magdalena E. Sobieszczyk, Yaoxing Huang, Kwok-Yung Yuen, Harris H. Wang, Zizhang Sheng, David D. Ho

**Affiliations:** 1 Aaron Diamond AIDS Research Center, Columbia University Vagelos College of Physicians and Surgeons, New York, NY USA; 2 Department of Microbiology and Immunology, Columbia University Vagelos College of Physicians and Surgeons, New York, NY USA; 3 Department of Systems Biology, Columbia University Vagelos College of Physicians and Surgeons, New York, NY USA; 4 State Key Laboratory of Emerging Infectious Diseases, Carol Yu Centre for Infection, Department of Microbiology, School of Clinical Medicine, Li Ka Shing Faculty of Medicine, University of Hong Kong, Hong Kong Special Administrative Region, China; 5 Centre for Virology, Vaccinology and Therapeutics, Hong Kong Science and Technology Park, Hong Kong Special Administrative Region, China; 6 Division of Infectious Diseases, Department of Medicine, Columbia University Vagelos College of Physicians and Surgeons, New York, NY USA; 7 Department of Pathology and Cell Biology, Columbia University Vagelos College of Physicians and Surgeons, New York, NY USA

**Keywords:** SARS-CoV-2, Vaccines, Viral immune evasion, Antiviral agents

## Abstract

The identification of the Omicron (B.1.1.529.1 or BA.1) variant of severe acute respiratory syndrome coronavirus 2 (SARS-CoV-2) in Botswana in November 2021^1^ immediately caused concern owing to the number of alterations in the spike glycoprotein that could lead to antibody evasion. We^2^ and others^3–6^ recently reported results confirming such a concern. Continuing surveillance of the evolution of Omicron has since revealed the rise in prevalence of two sublineages, BA.1 with an R346K alteration (BA.1+R346K, also known as BA.1.1) and B.1.1.529.2 (BA.2), with the latter containing 8 unique spike alterations and lacking 13 spike alterations found in BA.1. Here we extended our studies to include antigenic characterization of these new sublineages. Polyclonal sera from patients infected by wild-type SARS-CoV-2 or recipients of current mRNA vaccines showed a substantial loss in neutralizing activity against both BA.1+R346K and BA.2, with drops comparable to that already reported for BA.1 (refs. ^2,3,5,6^). These findings indicate that these three sublineages of Omicron are antigenically equidistant from the wild-type SARS-CoV-2 and thus similarly threaten the efficacies of current vaccines. BA.2 also exhibited marked resistance to 17 of 19 neutralizing monoclonal antibodies tested, including S309 (sotrovimab)^7^, which had retained appreciable activity against BA.1 and BA.1+R346K (refs. ^2–4,6^). This finding shows that no authorized monoclonal antibody therapy could adequately cover all sublineages of the Omicron variant, except for the recently authorized LY-CoV1404 (bebtelovimab).

## Abstract (web summary)

A study reports on the antigenic characterization of SARS-CoV-2 BA.1, BA.1.1 and BA.2 and the neutralizing activity of different monoclonal antibodies and sera against them.

## Main

**Fig. 1:**
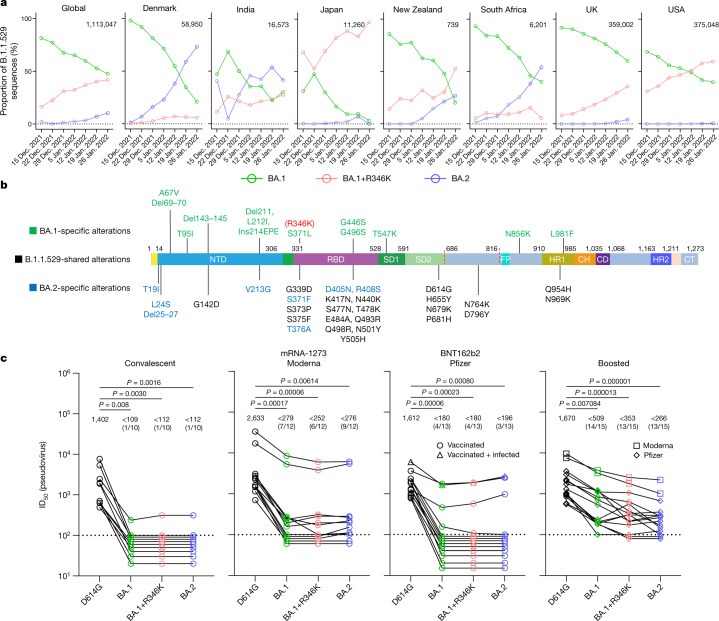
BA.2 exhibits a similar serum neutralization profile to those of BA.1 sublineages. **a**, Proportions of BA.1, BA.1+R346K and BA.2 in B.1.1.529 sequences on GISAID over the latter half of December 2021 and January 2022. The value in the upper right corner of each box denotes the cumulative number of Omicron sequences. **b**, Alterations in the B.1.1.529 lineage. NTD, N-terminal domain; RBD, receptor-binding domain; SD1, subdomain 1; SD2, subdomain 2; FP, fusion peptide; HR1, heptad repeat 1; CH, central helix; CD, connector domain; HR2, heptad repeat 2; CT, cytoplasmic tail. **c**, Pseudovirus neutralization by convalescent and vaccinee sera. *n* = 10, 12, 13 and 15 biologically independent serum samples, respectively, for convalescent, mRNA-1273, BNT162b2 and boosted groups. The values above the points indicate geometric means. The numbers in parentheses denote the numbers of samples above the limit of detection (LOD) of 100. Values below the LOD are arbitrarily plotted to allow for visualization of each sample. *P* values were determined by a two-sided Friedman test followed by Dunn’s multiple comparisons test.

The rise of the Omicron (B.1.1.529) variant to become the dominant variant of SARS-CoV-2 globally has been remarkable^8^. Continuing surveillance of its evolution in the population in December 2021 and January 2022 has revealed that the proportion of the original form, BA.1, has been decreasing steadily whereas the proportions of two other sublineages have increased noticeably (Fig. 1a). In fact, the BA.1+R346K sublineage now accounts for about 40% of Omicron sequences globally, and about 35–60% in New Zealand, the UK and the USA. On the other hand, the BA.2 sublineage accounts for only about 10% of Omicron sequences globally, but it is not only on the rise but also the dominant form in countries such as Denmark, India and South Africa. These three sublineages of Omicron share 21 alterations in the spike protein, wherein BA.2 contains 8 unique alterations and BA.1 contains 13 unique alterations (Fig. 1b). Of course, BA.1+R346K has one alteration more than BA.1. Given these differences, their antigenic properties cannot be assumed to be the same or similar.

## Serum neutralization of sublineages

**Extended Data Fig. 1:**
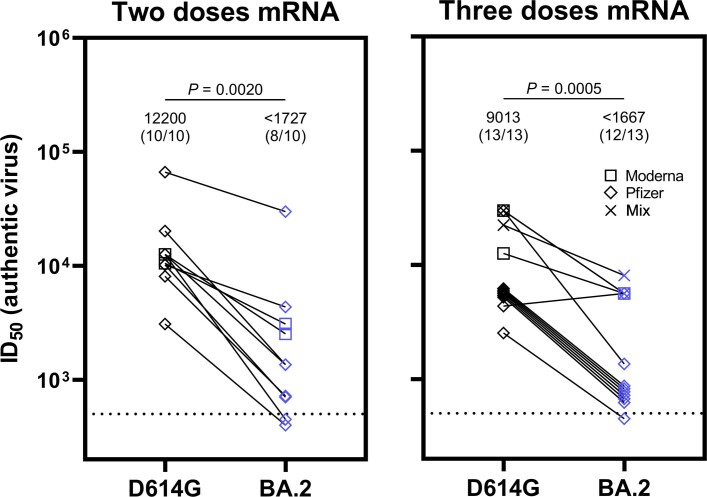
Serum neutralization of authentic viruses. Authentic virus neutralization by vaccinee sera. *n* = 10 and 13 biologically independent serum samples, respectively, for two doses mRNA and three doses mRNA groups. Values above points indicate the geometric mean. Numbers in parentheses denote the number of samples above the limit of detection (LOD) of 500. Values below the LOD and those that overlap are plotted to allow for visualization of each sample. P values were determined by two-sided Wilcoxon matched-pairs signed rank test.

Therefore, we first investigated the sensitivity of the Omicron sublineages to neutralization by polyclonal sera from convalescent individuals or individuals given mRNA vaccines, with or without a booster shot. These serum samples, as well as the pseudovirus neutralization assay used, were identical to ones previously reported^2^. The wild-type D614G pseudovirus was included as a comparator. As was observed and reported for BA.1 (refs. ^2,3,5,6^), a marked and significant loss of neutralizing activity of the serum against BA.1+R346K and BA.2 relative to D614G was noted, with neutralizing titres for numerous samples dropping below the limit of detection (Fig. 1c). The loss of neutralizing activity against BA.1+R346K or BA.2 sublineages was less prominent for sera obtained from individuals who received a booster vaccination (Fig. 1c, right panel), consistent with reported findings for BA.1 (refs. ^2,3,6^). Among these samples, the mean serum neutralizing titres against Omicron sublineages were significantly lower than the mean titre for D614G; although the mean titre was slightly lower for BA.2, the difference from that of the BA.1 sublineages did not reach statistical significance (*P* = 0.242). Finally, we confirmed the pseudovirus neutralization data by testing a separate set of sera from individuals given mRNA vaccines for neutralization of authentic viruses (Extended Data Fig. 1 and Extended Data Table 1). As above, neutralizing titres dropped significantly against authentic BA.2 virus relative to D614G.

## Antibody neutralization of sublineages

**Fig. 2:**
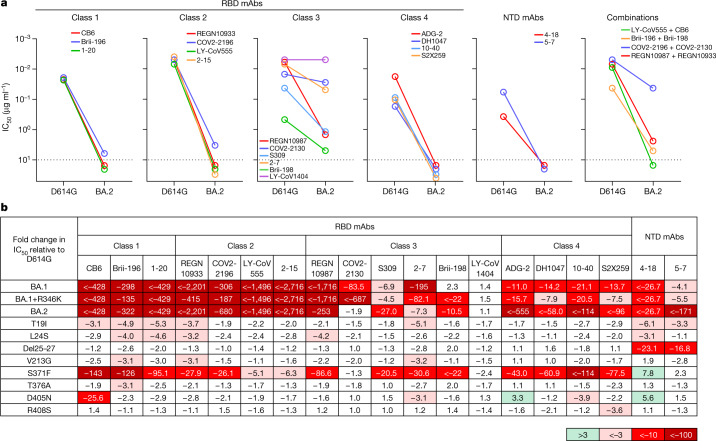
BA.2 differs in resistance profile to monoclonal antibodies. **a**, Pseudovirus neutralization by monoclonal antibodies (mAbs). Values above the LOD of 10 μg ml^−1^ (dotted line) are arbitrarily plotted to allow for visualization of each sample**. b**, Fold change in IC_50_ values relative to D614G of neutralization of Omicron variants, as well as point mutants unique to BA.2.

To further examine antigenic differences in the spike protein of these Omicron sublineages, a panel of 19 neutralizing monoclonal antibodies was used as probes. Seventeen were directed to different epitope clusters (classes 1–4) in the receptor-binding domain (RBD), whereas two were directed to the N-terminal domain (NTD). These antibodies included REGN10987 (imdevimab)^9^, REGN10933 (casirivimab)^9^, COV2-2196 (tixagevimab)^10^, COV2-2130 (cilgavimab)^10^, LY-CoV555 (bamlanivimab)^11^, CB6 (etesevimab)^12^, Brii-196 (amubarvimab)^13^, Brii-198 (romlusevimab)^13^, S309 (sotrovimab)^7^, LY-CoV1404 (bebtelovimab)^14^, ADG-2 (ref. ^15^), DH1047 (ref. ^16^) and S2X259 (ref. ^17^), as well as 1-20, 2-15, 2-7, 4-18, 5-7 (ref. ^18^) and 10-40 (ref. ^19^) from our group. Overall, 17 of the 19 monoclonal antibodies were either totally inactive or severely impaired in neutralizing BA.2 (Fig. 2a), similar to previous findings for BA.1 and BA.1+R346K (ref. ^2^) but with important differences (Fig. 2b). All class 4 antibodies tested lost greater neutralizing potency against BA.2 versus BA.1 sublineages. Two class 3 antibodies, COV2-2130 and 2-7, retained decent activity against BA.2 but had almost no activity against BA.1 viruses. S309 or sotrovimab lost 27-fold neutralizing activity against BA.2; this is important because it is an authorized monoclonal antibody that was found to retain activity against the original form of Omicron^2–4^. LY-CoV1404, the most recently authorized monoclonal antibody, remained potent in neutralizing all Omicron sublineages, suggesting that there is still a patch in this antibody-binding region that is unaffected by all spike alterations found in SARS-CoV-2 variants so far. Although there was a lack of an observable difference among the Omicron sublineages in neutralization by polyclonal sera (Fig. 1c), important antigenic differences do exist when probed by monoclonal antibodies. BA.1 seems to be more resistant to class 3 antibodies than BA.2 (except for S309), whereas BA.2 is more resistant to all class 4 antibodies tested. Our recent study^2^ showed that previous SARS-CoV-2 variants, such as Beta (B.1.351) and Delta (B.1.617.2), evolved to resist class 1, class 2 and NTD antibodies first, and then the Omicron variant seemingly has further evolved to resist class 3 and class 4 antibodies in addition. Our current findings suggest that the Omicron sublineages may have diverged under slightly different pressure from class 3 and class 4 antibodies to the RBD.

## Alterations conferring antibody resistance

Finally, we constructed each of the eight BA.2-specific spike alterations alone as pseudoviruses and tested them using the same panel of 19 monoclonal antibodies (Fig. 2b). S371F broadly affected most of the RBD-directed antibodies, similar to what was observed for S371L in BA.1 (ref. ^2^) but with a greater negative impact, perhaps due to the bulkier side chain of phenylalanine. Notably, S371F seems to be responsible for the loss in potency of S309, although this alteration was not observed previously as a marker for clinical resistance to sotrovimab^20^. CB6 was adversely affected by the D405N alteration, probably owing to its position in the epitope of this antibody^12^. It is not clear how T19I and L24S alterations in the NTD subtly impaired the neutralizing activity of class 1 antibodies to the RBD.

## Discussion

In summary, we have comprehensively evaluated the antigenic properties of two sublineages of the Omicron variant, BA.1+R346K and BA.2, and we believe that our results have important clinical implications. First, polyclonal sera showed a substantial loss in neutralizing activity against both sublineages, with drops comparable to that against BA.1 (Fig. 1c). These three sublineages of Omicron, therefore, seem to be antigenically equidistant from the wild-type SARS-CoV-2, probably threatening the efficacies of current coronavirus disease 2019 (COVID-19) vaccines to a similar extent. The present study, however, does not address the antigenic distance between BA.1 and BA.2, the determination of which will require cross-neutralization experiments using sublineage-specific sera. Second, monoclonal antibodies were affected in a disparate manner for the different Omicron sublineages. For clinically approved or authorized antibodies, S309 (sotrovimab) retained activity against both BA.1 and BA.1+R346K, but its activity against BA.2 has dropped 27-fold (Fig. 2b) to a 50% inhibitory concentration (IC_50_) of about 1 μg ml^−1^ (Fig. 2a). COV2-2130 (cilgavimab) and its combination with COV2-2196 (tixagevimab) retained activity against BA.2, but this antibody combination is authorized only for preventive use. Only the recently authorized LY-CoV1404 (bebtelovimab) could adequately treat all sublineages of the Omicron variant. As COVID-19 treatment options are narrowed by the emergence of more and more variants, it is imperative that we continue to devise novel strategies to contain this ever-evolving pathogen.

## Methods

### Data reporting

No statistical methods were used to predetermine sample size. The experiments were not randomized and the investigators were not blinded to allocation during experiments and outcome assessment.

### Serum samples

For the pseudovirus neutralization experiments, identical samples from a previous study were utilized^2^. For the authentic virus neutralization experiments, the samples are described in Extended Data Table 1. All collections were conducted under protocols reviewed and approved by the Institutional Review Board of Columbia University. All of the participants provided written informed consent.

### Antibodies

Antibodies were expressed as previously described^18^. Briefly, Vh and Vl genes for each antibody were codon optimized and synthesized (GenScript), and then inserted into mammalian expression vectors. These plasmids were transiently transfected into Expi293 cells (Thermo Fisher) using polyethylenimine and cultured for 5 days, and then the antibody was purified by affinity chromatography using rProtein A Sepharose (GE). REGN10933, REGN10987, COV2-2130 and COV2-2196 were provided by Regeneron Pharmaceuticals, Brii-196 and Brii-198 were provided by Brii Biosciences, and CB6 was provided by B. Zhang and P. Kwong (NIAID).

### Cells

Expi293 cells were obtained from Thermo Fisher (catalogue number A14527), Vero E6 cells were obtained from ATCC (catalogue number CRL-1586), HEK293T cells were obtained from ATCC (catalogue number CRL-3216), and Vero-E6-TMPRSS2 cells were obtained from JCRB (catalogue number JCRB1819). All cells were purchased from authenticated vendors and morphology was visually confirmed before use. All cell lines tested mycoplasma negative.

### Pseudovirus production

Spike expression constructs for variant SARS-CoV-2 spikes were produced by an in-house gene synthesis method as previously described^2^. Constructs were confirmed by sequencing, and then transfected into HEK293T cells using Lipofectamine 3000 (Thermo Fisher) according to the manufacturer’s instructions. Cells were washed 24 h post-transfection with complete medium (DMEM + 10% FBS + penicillin/streptomycin) and then infected with rVSV-G-pseudotyped ΔG-luciferase (G*ΔG-luciferase, Kerafast). Cells were thoroughly washed 2 h post-infection with complete medium, and then incubated for a further 24 h at 37 °C under 5% CO_2_. Pseudoviruses were then collected and incubated with anti-VSV-G hybridoma supernatant for 1 h at 37 °C (I1-Hybridoma, ATCC) to neutralize residual rVSV-G. The titre of each pseudovirus was determined by serially diluting the virus in complete medium in 96-well plates, and then incubating with 40,000 Vero E6 cells for approximately 12 h at 37 °C under 5% CO_2_. Following infection, luminescence was quantified using the Luciferase Assay System (Promega) according to the manufacturer’s instructions and measured with a SpectraMax i3x Multi-Mode Microplate Reader (Molecular Devices) using SoftMax Pro 7.0.2 (Molecular Devices), and then the titre was determined by comparison to control wells with cells alone. Pseudoviruses were aliquoted and stored at −80 °C until use.

### Pseudovirus neutralization assay

Neutralization assays were conducted in 96-well plates by serially diluting sera or antibodies in complete medium, starting at 1:100 dilution or 10 µg ml^−1^, respectively, and incubating with pseudoviruses for 1 h at 37 °C. Following incubation, 40,000 Vero E6 cells were added to each well, and further incubated for approximately 12 h at 37 °C under 5% CO_2_. Luminescence was quantified using the Luciferase Assay System according to the manufacturer’s instructions and measured with a SpectraMax i3x Multi-Mode Microplate Reader using SoftMax Pro 7.0.2. Neutralization was determined by comparison to control wells with cells alone and with virus alone. IC_50_ values were calculated by fitting a nonlinear five-parameter dose–response curve in GraphPad Prism version 9.2.

### Authentic virus isolation and propagation

SARS-CoV-2 variants D614G (GISAID: EPI_ISL_497840) and BA.2 (GISAID: EPI_ISL_9845731) were isolated from respiratory tract specimens of patients with COVID-19 in Hong Kong by J.F.-W.C., K.-Y.Y. and colleagues at the Department of Microbiology, The University of Hong Kong. The viruses were propagated in Vero-E6-TMPRSS2 cells and the sequence was confirmed by next-generation sequencing before use.

### Authentic virus neutralization assay

Vero-E6-TMPRSS2 cells were seeded in 96-well plates in complete medium overnight at 37 °C under 5% CO_2_ to establish a monolayer. The following day, sera were serially diluted starting at 1:500 dilution in 96-well plates in triplicate in DMEM + 2% FBS and then incubated with 0.01 MOI of either virus at 37 °C for 1 h. Afterwards, the mixture was overlaid onto cells and further incubated at 37 °C under 5% CO_2_ for approximately 72 h. Cytopathic effects were then visually assessed in all wells and scored as either negative or positive for infection by comparison to control uninfected or infected wells in a blinded manner. Neutralization curves and IC_50_ values were derived by fitting a nonlinear five-parameter dose–response curve to the data in GraphPad Prism version 9.2.

### Reporting summary

Further information on research design is available in the Nature Research Reporting Summary linked to this paper.

## Online content

Any methods, additional references, Nature Research reporting summaries, source data, extended data, supplementary information, acknowledgements, peer review information; details of author contributions and competing interests; and statements of data and code availability are available at 10.1038/s41586-022-04594-4.

### Supplementary information

Reporting Summary

## Figures and Tables

**Extended Data Table 1:**
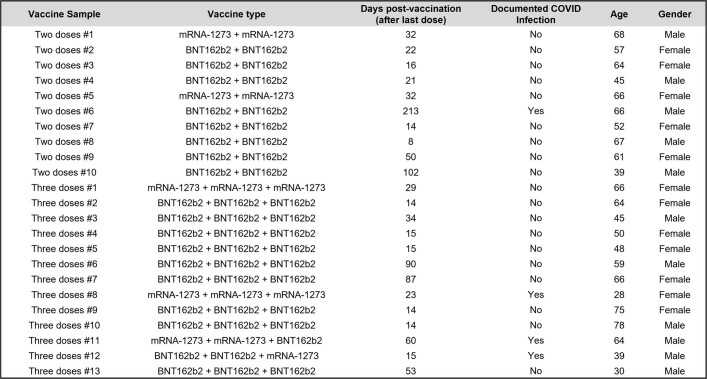
Demographics and vaccination information for serum samples from vaccinated individuals used in authentic virus neutralization experiments

## Data Availability

All experimental data are provided in the manuscript. Omicron prevalence analyses utilized sequences submitted to and available from GISAID (ref. ^8^). The sequences of the authentic viruses used in this study have been deposited to GISAID (https://www.gisaid.org/) under the accession numbers EPI_ISL_497840 (D614G) and EPI_ISL_9845731 (BA.2). Materials use in this study will be made available under an appropriate Materials Transfer Agreement.
