# Supplementary material for: Antibody evasion properties of SARS-CoV-2 Omicron sublineages
Source: Nature. 2022 Mar 3;604(7906):553–6. doi: 10.1038/s41586-022-04594-4 (PMC9021018; doi:10.1038/s41586-022-04594-4)
Supplement: Supplementary file 1 — Reporting Summary [file 41586_2022_4594_MOESM1_ESM.pdf]

nature portfolio

Corresponding author(s): David D. Ho

Last updated by author(s): Feb 23, 2022

Reporting Summary

Nature Portfolio wishes to improve the reproducibility of the work that we publish. This form provides structure for consistency and transparency in reporting. For further information on Nature Portfolio policies, see our Editorial Policies and the Editorial Policy Checklist.

Statistics

For all statistical analyses, confirm that the following items are present in the figure legend, table legend, main text, or Methods section.

n/a	Confirmed	
☐	☒	The exact sample size (*n*) for each experimental group/condition, given as a discrete number and unit of measurement
☐	☒	A statement on whether measurements were taken from distinct samples or whether the same sample was measured repeatedly
☐	☒	The statistical test(s) used AND whether they are one- or two-sided<br>*Only common tests should be described solely by name; describe more complex techniques in the Methods section.*
☒	☐	A description of all covariates tested
☒	☐	A description of any assumptions or corrections, such as tests of normality and adjustment for multiple comparisons
☐	☒	A full description of the statistical parameters including central tendency (e.g. means) or other basic estimates (e.g. regression coefficient) AND variation (e.g. standard deviation) or associated estimates of uncertainty (e.g. confidence intervals)
☐	☒	For null hypothesis testing, the test statistic (e.g. *F*, *t*, *r*) with confidence intervals, effect sizes, degrees of freedom and *P* value noted<br>*Give P values as exact values whenever suitable.*
☒	☐	For Bayesian analysis, information on the choice of priors and Markov chain Monte Carlo settings
☒	☐	For hierarchical and complex designs, identification of the appropriate level for tests and full reporting of outcomes
☒	☐	Estimates of effect sizes (e.g. Cohen's *d*, Pearson's *r*), indicating how they were calculated

Our web collection on statistics for biologists contains articles on many of the points above.

Software and code

Policy information about availability of computer code

Data collection	SoftMax Pro 7.0.2 (Molecular Devices, LLC) was used to measure luminescence in the pseudovirus neutralization assays.
Data analysis	GraphPad Prism (version 9.2) was used for data visualization and for statistical tests.

For manuscripts utilizing custom algorithms or software that are central to the research but not yet described in published literature, software must be made available to editors and reviewers. We strongly encourage code deposition in a community repository (e.g. GitHub). See the Nature Portfolio guidelines for submitting code & software for further information.

Data

Policy information about availability of data

All manuscripts must include a data availability statement. This statement should provide the following information, where applicable:

- Accession codes, unique identifiers, or web links for publicly available datasets
- A description of any restrictions on data availability
- For clinical datasets or third party data, please ensure that the statement adheres to our policy

All experimental data are provided in the manuscript. Omicron prevalence analyses utilized sequences submitted to and available from GISAID. The sequences of the authentic viruses used in this study are available at GISAID under accession numbers EPI_ISL_497840 (D614G) and EPI_ISL_9845731 (BA.2).

Field-specific reporting

Please select the one below that is the best fit for your research. If you are not sure, read the appropriate sections before making your selection.

☒ Life sciences ☐ Behavioural & social sciences ☐ Ecological, evolutionary & environmental sciences

For a reference copy of the document with all sections, see nature.com/documents/nr-reporting-summary-flat.pdf

Life sciences study design

All studies must disclose on these points even when the disclosure is negative.

Sample size	We used analogous sample sizes as in previous work (e.g. Wang et al 2021, Nature, Liu et al 2021, Nature), which we had previously determined to be sufficient sample sizes for comparisons between groups for these experiments.
Data exclusions	No data were excluded.
Replication	The key results, the serum neutralization of D614G and BA.2 (both in pseudoviruses and authentic viruses), and the neutralization of all of the viruses in Fig. 2b by S309, were repeated twice independently in technical triplicate with similar results. The results that are shown are representative. Other experiments were conducted in technical triplicate and not repeated, as these results were consistent with bioinformatic and structural analyses.
Randomization	As this is an observational study, randomization is not relevant.
Blinding	As this is an observational study, investigators were not blinded.

Reporting for specific materials, systems and methods

We require information from authors about some types of materials, experimental systems and methods used in many studies. Here, indicate whether each material, system or method listed is relevant to your study. If you are not sure if a list item applies to your research, read the appropriate section before selecting a response.

Materials & experimental systems

n/a	Involved in the study	
☐	☒	Antibodies
☐	☒	Eukaryotic cell lines
☒	☐	Palaeontology and archaeology
☒	☐	Animals and other organisms
☐	☒	Human research participants
☒	☐	Clinical data
☒	☐	Dual use research of concern

Methods

n/a	Involved in the study	
☒	☐	ChIP-seq
☒	☐	Flow cytometry
☒	☐	MRI-based neuroimaging

Antibodies

Antibodies used	All of the antibodies used in this study were produced in our laboratory or received from other laboratories. 1-20, LY-CoV555, 2-15, S309, 2-7, LY-CoV1404, ADG-2, DH1047, 10-40, S2X259, 4-18, and 5-7 were expressed and purified in-house as described previously in Liu et al 2020, Nature and in the Methods section of this manuscript. REGN10987, REGN10933, COV2-2196, and COV2-2130 were produced and provided by Regeneron Pharmaceuticals, Brii-196 and Brii-198 were produced and provided by Brii Biosciences, CB6 was produced and provided by Baoshan Zhang and Peter Kwong (NIAID).
Validation	All of the antibodies except LY-CoV1404 have been validated in previous studies both by binding to SARS-CoV-2 spike and neutralization of SARS-CoV-2 (both pseudovirus and authentic virus), and when applicable, have been confirmed to give similar results as that described in publications by other groups. Specifically, 1-20 and 4-18 were tested in Liu et al 2020, Nature, CB6, Brii-196, 910-30, REGN10933, COV2-2196, LY-CoV555, 2-15, REGN10987, COV2-2130, S309, 2-7, Brii-198, and 5-7 were tested in Wang et al 2021, Nature, and ADG-2, DH1047, 10-40, and S2X259 were tested in Liu et al 2021, bioRxiv. LY-CoV1404 was newly produced and tested prior to use in this study and confirmed to have similar results as that of the original publication from which it is derived (Westendorf et al 2022, bioRxiv).

Eukaryotic cell lines

Policy information about cell lines

Cell line source(s)	Expi293 cells were obtained from Thermo Fisher (Catalog #A14527), Vero E6 cells were obtained from ATCC (Catalog

Cell line source(s)	#CRL-1586), HEK293T cells were obtained from ATCC (Catalog #CRL-3216), and Vero-E6-TMPRSS2 cells were obtained from JCRB (Catalog #JCRB1819).
Authentication	Cell lines were purchased from authenticated vendors, and morphology was also confirmed visually prior to use.
Mycoplasma contamination	Cell lines tested mycoplasma negative.
Commonly misidentified lines (See ICLAC register)	No commonly misidentified cell lines were used in this study.

Human research participants

Policy information about studies involving human research participants

Population characteristics	Population characteristics for the sera utilized in the pseudovirus neutralization assays are described in Extended Data Table 1 of Liu et al 2021, Nature (ref 2). Convalescent samples had the following ranges: 9-120 days post-symptoms, 45-79 years old, 4/10 female, 6/10 male. We presume all of these individuals were infected with the wild-type strain of SARS-CoV-2 as these samples were collected in Spring of 2020. Vaccinee samples had the following ranges: 6-213 days post-vaccination, 26-78 years old, 12/40 two mRNA-1273 vaccinations, 13/40 two BNT162b2 vaccinations, 2/40 three mRNA-1273 vaccinations, 13/40 three BNT162b2 vaccinations, 1/40 previously infected, 39/40 uninfected, 20/40 female, 20/40 male.<br><br>Population characteristics for the vaccinee sera utilized in the authentic virus neutralization assays are described in Extended Data Table 1 of this manuscript. These samples had the following ranges: 8-213 days post-vaccination, 28-78 years old, 2/23 two mRNA-1273 vaccinations, 8/23 two BNT162b2 vaccinations, 2/23 three mRNA-1273 vaccinations, 9/23 three BNT162b2 vaccinations, 1/23 two mRNA-1273 vaccinations followed by one BNT162b2 vaccination, 1/23 two BNT162b2 vaccination followed by one mRNA-1273 vaccination, 4/23 previously infected, 19/23 uninfected, 12/23 female, 11/23 male.
Recruitment	For convalescent sera, convalescing patients volunteered and were enrolled in an observational cohort study at Columbia University Irving Medical Center in Spring of 2020. For the vaccinee sera, individuals volunteered and were enrolled in an observational cohort study at Columbia University Irving Medical Center to study the immunological responses to SARS-CoV-2 in individuals who had received COVID-19 vaccines. Self-selection biases may have affected the demographics of the enrolled population, but are not expected to have impacted the results of this study. High titer samples were specifically chosen within each of the serum groups so that fold-change in titer could be better determined.
Ethics oversight	All collections were conducted under protocols reviewed and approved by the Institutional Review Board of Columbia University. All of the participants provided written informed consent.

Note that full information on the approval of the study protocol must also be provided in the manuscript.
